# The Effect of Carpal Tunnel Release on Neuropathic Pain in Carpal Tunnel Syndrome

**DOI:** 10.1155/2017/8098473

**Published:** 2017-09-10

**Authors:** Motoki Sonohata, Toshiyuki Tsuruta, Hiroko Mine, Akihiko Asami, Hideki Ishii, Kenji Tsunoda, Masaaki Mawatari

**Affiliations:** ^1^Department of Orthopaedic Surgery, Faculty of Medicine, Saga University, 5-1-1 Nabeshima, Saga 849-8501, Japan; ^2^Tsuruta Orthopaedic Clinic, No. 1241-6, Katsu, Ushizu-cho, Ogi City, Saga 849-0306, Japan; ^3^Department of Orthopaedic Surgery, Saga Insurance Hospital, Saga 849-8522, Japan; ^4^Sakaemachi Orthopaedic Clinic, 2-8 Sakaemachi, Saga City, Saga 840-0803, Japan

## Abstract

**Purpose:**

The aim of this study was to determine the risk factors of neuropathic pain (NP) in the patient with carpal tunnel syndrome (CTS) before and after the carpal tunnel release.

**Materials and Methods:**

One hundred and two CTS patients were enrolled in the study. The pain score was measured by the visual analogue score. NP was determined by the painDETECT (PD) questionnaire. All subjects were divided into 3 groups at 12 weeks after surgery: an Improved, Unchanged, and Worsened group. The risk factors of worsening NP after surgery were evaluated.

**Results:**

We found that 36% and 18% of patients with CTS had neuropathic pain before and 12 weeks after surgery, respectively, and pain was significantly stronger than in those without NP. The PD score of eight hands worsened after surgery. In the “Improved group,” the average age at the surgery was younger and the pain score was lower than in the “Unchanged group.”* Conclusions.* The surgery was very effective on NP of CTS; however, the PD in 7% of hands worsened after surgery. Risk factors before surgery that predicted worse NP after surgery were found to be a younger age, weaker pain, and the absence of night pain.

## 1. Introduction

Carpal tunnel syndrome (CTS) is the most common entrapment neuropathy of the median nerve at the wrist [1]. In the general population, CTS is regarded as a common disease, and the prevalence of CTS is estimated to be 2.7% as confirmed by clinical and electrophysiological findings [2]. CTS has been associated with trauma, diabetes, rheumatoid arthritis, acromegaly, hypothyroidism, and pregnancy. It has also been associated with vibration and certain activities involving repetitive and forceful movements of the hands [3]. Typical symptoms of CTS are numbness and paresthesia in the thumb, index, middle finger, and the radial half of the ring finger. The numbness and the paresthesia are often exacerbated at night. As the disease progresses, the thenar muscle becomes atrophic and weakens. Most patients with CTS are treated by splinting, oral drugs, steroid injections, and decompression surgery, including open or endoscopic carpal tunnel release [1, 4]. Decompression surgery by carpal tunnel release is a minimally invasive and very effective procedure [1]. However, symptomatic relief with conservative treatment has been less than satisfactory, and surgical decompression, often considered the definitive solution, yields good results in only 75% of cases [5]. The standard treatments for CTS fail to result in complete satisfaction. In open carpal tunnel release, pillar pain, which is thought to be caused by injury to the palmar branch of median nerve (essentially neuropathic pain), is one of the most commonly reported reasons for pain after carpal tunnel release [6]. However, even with endoscopic carpal tunnel release, some patients fail to achieve complete satisfaction with their outcomes.

Pain is classified into nociceptive pain and neuropathic pain (NP). Damage or dysfunction of the central or peripheral nervous system induces the development of NP [7, 8]. Several tools have been developed to measure NP in patients with chronic pain [9–11]. A number of different diseases have been found to cause NP [7, 8, 12, 13], including CTS [14–16], which frequently manifests with NP [14–16].

However, only a few papers have described changes in NP before and after treatment for several diseases. In patients with shoulder impingement syndrome, those with high preoperative levels of central sensitization, often induced by NP, had a poor outcome after surgery [12]. However, the efficacy of carpal tunnel release in relieving NP in patients with CTS has not been reported.

The aim of this study was to evaluate NP in patients with CTS and the change in NP after surgery and to identify risk factors for persistent NP after surgery through an observational consecutive clinical study.

This study was approved by the Institutional Review Board (IRB) of Tsuruta Orthopaedic Clinic. Informed consent was obtained from each patient.

## 2. Materials and Methods

The subjects of this study consisted of consecutive patients that were newly diagnosed with idiopathic CTS in December 2011 and December 2014 at a single center (Tsuruta Orthopaedic Clinic, Saga, Japan).

CTS was diagnosed based on the clinical and electrophysiological findings, and 425 hands were nominated as having CTS. The exclusive criteria for distinguishing idiopathic from secondary carpal tunnel disease include the presence of trauma or rheumatoid arthritis, being in the perinatal period, and being on hemodialysis. One hundred and nine hands in 102 patients who completely answered the questionnaire with over 12 weeks of follow-up were incorporated into the current study (Table 1).

The distal latency of the median nerve in electrophysiological study was measured using the Neuropack U device (NIHON KOHDEN, Tokyo, Japan). For the electrophysiological measurement, the distal latency of the median nerve was induced at the abductor pollicis brevis 7 cm distal from the stimulated point by stimulating the median nerve halfway between the palmaris longus and flexor carpi radialis. A pinch gauge (FUJI SEIKO Co., Ltd., Chiba, Japan) was used to measure the power of the tip pinch between the thumb, index finger, and little finger. A Manual Muscle Testing (MMT) system was used to measure the muscle power of the abductor pollicis brevis [17]. The MMT consists of 6 levels: 5, normal power; 4, good; 3, fair; 2, poor; 1, trace; and 0, zero. The Semmes-Weinstein Monofilaments test (SAKAI Med, Tokyo, Japan) was used to measure the sensory disturbance.

All patients completed the Visual Analogue Scale (VAS) examination and painDETECT (PD) [9] screening based on their assessment of their NP. The VAS was assessed on a 100 mm long horizontal line. The patients were informed that the left end of the scale represented “no pain” and that the right end represented the “most severe pain imaginable.” They were then instructed to mark the intensity of the pain they were currently experiencing on the line. The distance between the 0 mm mark and the placement of the patient's mark was measured to obtain a numeric interpretation of their pain. The PD comprises seven items for evaluating the pain quality, one for evaluating the pain pattern, and one for evaluating the pain radiation, all of which contribute to an aggregate score (range: −1 to 38). The PD has been validated against an expert physician diagnosis of NP in people with a range of chronic pain conditions, including “typical” NP and “typical” non-NP settings. Patients were divided into three groups: likely (PD score ≥ 19), possible (score 13–18), and unlikely to have NP (score ≤ 12). The VAS was assessed at three time points for all patients: the pain at entry, the most severe pain during the subsequent four weeks, and the average pain during the four weeks. The exacerbation of pain at night (night pain) was also evaluated using the VAS score. All of the above factors were determined before and at 4, 8, and 12 weeks after surgery.

The patients were also asked to fill out the Japanese Society for Surgery of the Hand version of the Disability of Arm, Shoulder, and Hand questionnaire (DASH-JSSH) [18] in order to assess the function of the upper limb. The DASH is a suitable modality for measuring the health status outcome because it is mainly used as a measure of disability [19]. This 30-item scale focuses on a patient's upper extremities [20]. Each item has five response choices (Likert scale of 1 to 5), ranging from “no difficulty or no symptoms” (score of 1) to “unable to perform activity or very severe symptoms” (score of 5). The items ask about the severity of each of the symptoms of pain, activity-related pain, tingling, weakness, and stiffness (5 items, numbers 24–28); the degree of difficulty when performing various physical activities because of an arm, shoulder, or hand problem (21 items, numbers 1–21); the effect of the upper extremity problem on social activities, work, and sleep (3 items, numbers 22, 23, and 29); and the psychological effect on their self-image (1 item, number 30). The sum of these scores with transformation provides the DASH disability/symptom (DASHDS) score, which ranges from 0 (no disability) to 100 (the severest disability).

The subjects were divided into 3 groups at 12 weeks after surgery: an Improved, Unchanged, and Worsened group. The “Improved group” included any hands going from likely to possible or unlikely, or from possible to unlikely. The “Worsened group” included any hands going from unlikely to likely or possible, or from possible to likely.

The rest of the patients were designated as the “Unchanged group.”

All statistical analyses were performed using the SPSS software program for Windows (Version 22; IBM Corp, Armonk, NY, USA). The mean age, disease duration, distal latency, S-W test, MMT of APB, tip pinch, grip power, pain score (VAS), and DASH score between two groups were compared using Student's* t*-test. The male-to-female ratio and the existence-to-nonexistence of night pain ratio were compared using the *χ*^2^ test. The proportions of Unlikely NP versus Possible or Likely NP before and after surgery were compared using the McNemar test. Statistical significance was set at *p* < 0.05. Several factors were compared between the Improved, Unchanged, and Worsened groups at 12 weeks after surgery using an analysis of variance (ANOVA) followed by Scheffe's test.

## 3. Results

The characteristics of the patients are shown in Table 1. The current study included 109 hands in 102 patients (39 male, 63 female, mean age of 69.3 years, and an average duration of symptoms of 19.5 months). The findings of the examination before surgery are shown in Table 2. Regarding the electrophysiological findings, the average distal latency was 10.1 msec, and the minimum distal latency was 3.5 msec. Regarding the VAS at entry point, 61 hands (56%) had a VAS < 30 (Table 2).

Table 3 shows the changes in the PD score before and at 12 weeks after surgery. A total of 36% of CTS hands were suggestive of having NP (possible or likely to have NP). At 12 weeks after surgery, 18% of hands were still suggestive of having NP (possible or likely to have NP). Twelve hands with Possible NP at 12 weeks after surgery included 2 Unlikely NP, 8 Possible NP, and 2 Likely NP before surgery. Eight hands with Likely NP at 12 weeks after surgery included 3 Unlikely NP, 3 Possible NP, and 2 Likely NP before surgery. The findings on comparing the proportions of Unlikely versus Possible/Likely NP before and after surgery were as follows: before versus 4 weeks after operation (*p* < 0.01), before versus 8 weeks after operation (*p* < 0.01), before versus 12 weeks after operation (*p* < 0.01), 4 versus 8 weeks after operation (*p* = 0.063), 4 versus 12 weeks after operation (*p* < 0.01), and 8 versus 12 weeks after operation (*p* = 0.256). Therefore, there were significantly fewer patients with NP at 4, 8, and 12 weeks after surgery than with NP before surgery. Furthermore, a significant reduction in the number of patients with NP after surgery occurred at 8 weeks after surgery.

Table 4 shows the relationship between the three groups of PD (Unlikely NP, Possible NP, and Likely NP) and the clinical characteristics before surgery. In the MMT of tip pinch and grip power, those with “Likely NP” had a significantly weaker power than those with “Likely NP.” In all pain scores, those with “Unlikely NP” were significantly weaker than those with “Possible NP” and/or “Likely NP”.

Table 5 shows the relationship between the painDETECT scores at 12 weeks after surgery and the clinical characteristics. In the data before surgery, there was a significant difference in only the average pain during four weeks between “Unlikely NP” and “Possible NP.” At 12 weeks after surgery, in all pain scores, those with “Unlikely NP” were significantly weaker than those with “Possible NP” and “Likely NP.”

Table 6 shows a comparison between the Improved, Unchanged, and Worsened groups at 12 weeks after surgery. The average age in the “Improved group” was older than that in the “Unchanged group” (*p* < 0.05). Before surgery, all pain scores except for night pain in the “Improved group” were higher than those in the “Unchanged group” (all *p* < 0.01). However, there was a significant difference in the rate of existence of night pain between these two groups. At 12 weeks after surgery, night pain in the “Worsened group” was significantly stronger than in the “Improved group” or “Unchanged group.”

No significant differences were noted in the DASH score before or at 12 weeks after surgery in any groups.

## 4. Discussion

Carpal tunnel release is the most common surgical treatment for CTS, and many symptoms of CTS are improved after surgery; however, surgery fails to completely resolve symptoms in some patients [21]. The risk factors for inadequate recovery or even condition degradation after surgery are unclear. This study was the first to investigate the effect of open carpal tunnel release on NP in CTS patients.

CTS is one of the most common causes of NP because it is the major entrapment neuropathy [1]. However, in the present study, 38 (28%), and 9 (8%) hands were considered to either possibly or likely have NP before surgery. These results may be due to either of two reasons: First, the patients with CTS may have visited the clinic with a chief complaint of numbness, not pain. Typically, the first symptom of CTS is waking at night with numbness and pain in the median nerve distribution and aggravation of these symptoms by activities. In the current study, the average disease duration was 19.5 months, so the patients did not visit the clinic during the early phase of CTS [22].

Second, the PD might have issues concerning the indication, as it was initially developed to assess back pain [9]. The appropriateness of using the PD to assess pain due to non-back-related disease is not clear. Therefore, if we had used other screening tools, we might have obtained different results. The Pain Quality Assessment Scale (PQAS) [23] may have been more suitable for the current study; however, this was a retrospective study, so this was not possible.

Generally, the pain level of NP is larger than the pain level of nociceptive pain and may cause chronic pain [24]. We noted significant differences in all pain scores, grip power, and tip pinch between the hands unlikely to have NP and those possible or likely to have NP before surgery (Table 4). Whether the main reason for the motor weakness was pain or thenar muscle disturbance is unclear. However, thenar muscle weakness does not directly affect the grip power or tip pinch (thumb-index). Therefore, the weakness of the grip power and tip pinch (thumb-index) was likely due to NP.

The proportion of CTS patients with night pain is significantly higher in those with NP than in those without NP before surgery [16]. However, we noted no significant difference in the rate of night pain before surgery among the three groups of NP at 12 weeks after surgery (Table 5). This finding suggests that night pain with NP responds well to carpal tunnel release. There were no significant differences in the distal latency between the unlikely to have NP group and the possible or likely to have NP group before and after surgery. Therefore, the motor weakness may have increased due to strong pain. At 12 weeks after surgery, pain scores were found to be significantly related to NP, similar to the results before surgery. In previous reports, NP affected the function of the upper limbs [12, 15], but there were no significant differences in the DASH score before and after surgery in any groups in the current study. If we had used other tools specifically designed for CTS as reported by Levine et al. [25], we might have observed a significant correlation between the ADL/QOL and NP.

Carpal tunnel release always affects the symptom of CTS. However, 8 hands (7%) were classified into the “Worsened group.” The values in the “Improved group” were higher than those in the “Unchanged group” for all pain scores except night pain before surgery. In addition, the hands in the “Improved group” had night pain at a higher rate than those in the other groups (Table 6). This result seems contradictory; however, the “Unchanged group” mainly included hands with Unlikely NP before surgery, and the “Improved group” consisted of hands with Possible or Likely NP before surgery.

In the present study, younger patients with CTS did not necessarily experience an improvement in their symptoms after carpal tunnel release. One of the reasons for this may be because the threshold of pain in older patients might have been higher than in young patients [26]. This suggests that the degree of NP might depend on the overall degree of pain. However, we cannot draw any clear conclusions on this point.

There are some guidelines concerning the pharmacological management of NP [27–29]. In these guidelines, pregabalin and gabapentin are recommended as first-line drugs. Pregabalin binds to the alpha-2-delta subgroup of calcium channels, thereby reducing excitatory neurotransmitter release and preventing hyperalgesia and central sensitization [30]. Some papers have examined the clinical effects of gabapentin on CTS; however, their results were inconclusive [31–33]. After determining whether or not patients with CTS have NP, investigating the clinical effectiveness of those drugs is very important. Therefore, further studies should be conducted in order to develop a suitable treatment strategy for CTS.

Several limitations associated with the present study warrant mention. First, the study group was relatively small. In particular, the 8 hands in the “Worsened group” may be too small sample size to demonstrate statistical significance. However, the current study is the first report to investigate the efficacy of carpal tunnel release on NP in patients with CTS. These findings should be investigated further in future preliminary studies. Second, we did not examine the relationship between NP and the patients' quality of life. However, the current study included the DASH score to assess the activity of daily life, and this score can be used in place of a quality of life assessment.

In conclusion, 36% of patients with CTS had NP, and their muscle strength was significantly weaker and their pain significantly stronger than in those without NP before surgery. At 12 weeks after open carpal tunnel release, 18% of patients (*n* = 20) had NP. No relationship between the DASH scores and NP was detected. Strong pain was found to be significantly associated with NP both before and after surgery. Risk factors before surgery predicting worse NP after surgery were younger age, weaker pain, and the absence of night pain. The absence of night pain and weak pain before surgery did not guarantee good postoperative results. Unfortunately, the current study could not clarify the mechanism underlying the relationship between various pains before surgery and the clinical results after surgery. In clinical settings, it is necessary to conduct intervention, including administering medication, according to the symptoms of each case.

## Figures and Tables

**Table 1:** The demographic characteristics of the study participants.

Number of patients	102
Number of hands	109
Sex	
Male (patients [hands])	39, 40
Female (patients [hands])	63, 69
Age, years (mean ± SD, range)	69.3 ± 9.4, 42–92
Disease duration, months (mean ± SD, range)	19.5 ± 25.8, 1–188

SD: standard deviation.

**Table 2:** Findings of the examination before surgery.

Distal latency, msec (mean ± SD)	10.1 ± 3.5
S-W test (mean ± SD)	4.6 ± 1.2
MMT of APB (mean ± SD)	3.6 ± 0.9
Tip pinch (thumb-index), Kgf (mean ± SD)	3.7 ± 1.7
Tip pinch (thumb-little), Kgf (mean ± SD)	1.1 ± 0.6
Grip power, Kg (mean ± SD)	18.5 ± 8.7
Night pain (existence, nonexistence) (*n* [%])	58 (53%), 51 (47%)
Pain score (VAS)	
At entry point (mean ± SD)	28.3 ± 27.8
Most severe pain during 4 weeks (mean ± SD)	49.3 ± 34.7
Average pain during 4 weeks (mean ± SD)	29.7 ± 27.4
Night pain (mean ± SD)	28.8 ± 32.2
DASH score (mean ± SD)	28.4 ± 20.3

SD: standard deviation; S-W test: Semmes-Weinstein Monofilaments test; MMT: Manual Muscle Testing; APB: abductor pollicis brevis; VAS: visual analogue scale; DASH: Disability of Arm, Shoulder, and Hand.

**Table 3:** Changes in the painDETECT score.

	Before operation	4 weeks after operation	8 weeks after operation	12 weeks after operation
Unlikely NP	70 (64)	81 (74)	86 (79)	89 (82)
(0–12) (*n* [%])				
Possible NP	30 (28)	22 (20)	18 (16)	12 (11)
(13–18) (*n* [%])				
Likely NP	9 (8)	6 (6)	5 (5)	8 (7)
(19–38) (*n* [%])				
Possible or Likely NP	39 (36)	28 (26)	23 (21)	20 (18)
(13–38) (*n* [%])				

NP, neuropathic pain.

**Table 4:** The relationship between the painDETECT scores and clinical characteristics before surgery.

The data before operation	painDETECT (score) (number of hands)		
	Unlikely NP (≤12) (*n* = 70)	Possible NP (13–18) (*n* = 30)	Likely NP (≥19) (*n* = 9)
Sex (male, female) (*n* [%])	25 (36), 45 (64)	12 (40), 18 (60)	3 (33), 6 (67)
Age, years (mean ± SD)	68.6 ± 9.0	69.3 ± 9.8	74.6 ± 10.2
Disease duration, months (mean ± SD)	17.9 ± 21.0	27.1 ± 36.0	6.9 ± 7.2
Distal latency, msec (mean ± SD)	10.1 ± 3.5	9.9 ± 3.4	10.5 ± 4.6
S-W test (mean ± SD)	4.6 ± 1.1	4.5 ± 1.3	5.0 ± 1.3
MMT of APB (mean ± SD)	3.5 ± 0.9	3.8 ± 0.6	3.2 ± 1.2
Tip pinch (thumb-index), Kgf (mean ± SD)	4.0 ± 1.5^*∗∗*^	3.6 ± 1.8	2.1 ± 1.6
Tip pinch (thumb-little), Kgf (mean ± SD)	1.2 ± 0.6^*∗*^	1.1 ± 0.8	0.6 ± 0.3
Grip power, Kg (mean ± SD)	20.4 ± 9.0^*∗∗*^	16.3 ± 7.2	11.5 ± 5.3
Pain score (VAS)			
At entry (mean ± SD)	17.5 ± 22.6^*∗∗ψψ*^	46.0 ± 25.9	52.9 ± 27.1
Most severe pain during 4 weeks (mean ± SD)	37.6 ± 35.3^*∗ψψ*^	70.2 ± 22.2	71.3 ± 17.9
Average pain during 4 weeks (mean ± SD)	19.9 ± 24.4^*∗∗ψψ*^	46.0 ± 25.7	51.6 ± 16.2
Night pain (mean ± SD)	21.3 ± 28.6^*ψψ*^	43.7 ± 35.4	37.2 ± 31.8
Night pain (existence, nonexistence) (*n* [%])	31 (44), 39 (56)^*φ*^	21 (70), 9 (30)	6 (67), 3 (33)
DASH score (mean ± SD)	30.6 ± 22.1	23.9 ± 16.1	25.8 ± 17.0

NP: neuropathic pain; SD: standard deviation; S-W test: Semmes-Weinstein Monofilaments test; MMT: Manual Muscle Testing; APB: abductor pollicis brevis; VAS: visual analogue scale; DASH: Disability of Arm, Shoulder, and Hand; ^*∗*^significantly different to Likely NP (*p* < 0.05); ^*∗∗*^significantly different to Likely NP (*p* < 0.01); ^*ψψ*^significantly different to Possible NP (*p* < 0.01); ^*φ*^*p* < 0.05 calculated by *χ*^2^ test.

**Table 5:** The relationship between the painDETECT scores at 12 weeks after surgery and clinical characteristics.

painDETECT (score) (number of hands)	Unlikely NP (≤12) (*n* = 89)	Possible NP (13–38) (*n* = 12)	Likely NP (≥19) (*n* = 8)
Sex (male, female) (*n* [%])	32 (36), 57 (64)	6 (50), 6 (50)	2 (25), 6 (75)
Age (mean ± SD)	69.9 ± 9.7	65.3 ± 7.1	67.9 ± 6.6
Disease duration, months (mean ± SD)	19.5 ± 26.4	25.1 ± 25.0	12.1 ± 19.5
*The data before surgery*			
Distal latency, msec (mean ± SD)	9.9 ± 3.5	10.5 ± 3.6	11.5 ± 4.0
S-W test (mean ± SD)	4.5 ± 1.1	5.2 ± 1.3	4.7 ± 1.6
MMT of APB (mean ± SD)	3.6 ± 0.9	3.4 ± 0.8	3.1 ± 1.0
Tip pinch (thumb-index), Kgf (mean ± SD)	3.9 ± 1.7	3.2 ± 1.5	2.9 ± 1.9
Tip pinch (thumb-little), Kgf (mean ± SD)	1.1 ± 0.6	0.9 ± 0.5	0.8 ± 0.5
Grip power, Kg (mean ± SD)	19.0 ± 8.8	15.6 ± 6.9	18.1 ± 10.1
Pain score ( VAS)			
At entry (mean ± SD)	25.5 ± 27.5	42.4 ± 29.3	37.5 ± 23.1
Most severe pain during 4 weeks (mean ± SD)	46.2 ± 36.3	68.1 ± 16.9	56.1 ± 28.1
Average pain during 4 weeks (mean ± SD)	26.3 ± 26.6^*∗*^	51.6 ± 26.3	35.0 ± 29.7
Night pain (mean ± SD)	27.9 ± 31.8	39.1 ± 35.3	23.4 ± 33.1
Night pain (existence, nonexistence) (*n* [%])	47 (53), 42 (47)	8 (67), 4 (33)	3 (38), 5 (62)
DASH score (mean ± SD)	28.7 ± 20.9	27.6 ± 18.8	26.5 ± 18.1
*The data at 12 weeks after surgery*			
Distal latency, msec (mean ± SD)	7.6 ± 4.0	7.0 ± 4.1	9.3 ± 3.9
S-W test (mean ± SD)	3.9 ± 1.0	4.0 ± 1.0	3.8 ± 1.3
MMT of APB (mean ± SD)	3.8 ± 0.7	3.8 ± 0.6	3.5 ± 0.5
Tip pinch (thumb-index), Kgf (mean ± SD)	4.0 ± 1.5^*∗*^	2.7 ± 1.6	2.6 ± 1.6
Tip pinch (thumb-little), Kgf (mean ± SD)	0.8 ± 0.5	0.6 ± 0.2	0.5 ± 0.4
Grip power, Kg (mean ± SD)	17.0 ± 6.9	12.7 ± 4.4	14.1 ± 5.7
Pain score ( VAS)			
At entry (mean ± SD)	7.3 ± 12.1^*∗∗ψψ*^	41.3 ± 24.3	32.1 ± 22.1
Most severe pain during 4 weeks (mean ± SD)	21.9 ± 23.0^*∗∗ψ*^	63.2 ± 25.6	48.0 ± 18.7
Average pain during 4 weeks (mean ± SD)	8.4 ± 12.6^*∗∗ψψ*^	41.9 ± 22.4	31.4 ± 20.2
Night pain (mean ± SD)	6.7 ± 14.8^*∗∗ψψ*^	35.6 ± 36.1	28.6 ± 21.3
Night pain (existence, nonexistence) (*n* [%])	22 (25), 67 (75)^*φφ*^	7 (58), 5 (42)	7 (88), 1 (12)
DASH score (mean ± SD)	22.2 ± 18.9	18.5 ± 16.4	25.7 ± 25.9

NP: neuropathic pain; SD: standard deviation; S-W test: Semmes-Weinstein Monofilaments test; MMT: Manual Muscle Testing; APB: abductor pollicis brevis; VAS: visual analogue scale; DASH: Disability of Arm, Shoulder, and Hand; ^*∗*^significantly different to Possible NP (*p* < 0.05); ^*∗∗*^significantly different to Possible NP (*p* < 0.01); ^*ψ*^significantly different to Likely NP (*p* < 0.05); ^*ψψ*^significantly different to Likely NP (*p* < 0.01); ^*φφ*^*p* < 0.01 calculated by *χ*^2^ test.

**Table 6:** A comparison of the Improved, Unchanged, and Worsened groups at 12 weeks after surgery.

	Improved group (*n* = 26)	Unchanged group (*n* = 75)	Worsened group (*n* = 8)
Sex (male, female) (*n* (%))	10 (38), 16 (62)	29 (39), 46 (61)	1 (13), 7 (87)
Age (mean ± SD)	73.3 ± 10.2^*∗*^	68.1 ± 8.9	67.2 ± 7.6
Disease duration (months) (mean ± SD)	25.8 ± 37.4	16.3 ± 20.1	29.6 ± 25.3
*The data before surgery*			
Distal latency (ms) (mean ± SD)	9.6 ± 3.7	10.2 ± 3.5	10.4 ± 4.1
S-W test (mean ± SD)	4.6 ± 1.3	4.6 ± 1.1	4.7 ± 1.6
MMT of APB (mean ± SD)	3.8 ± 0.8	3.5 ± 0.9	3.3 ± 0.9
Tip pinch (thumb-index) (Kgf) (mean ± SD)	3.4 ± 1.9	3.9 ± 1.6	3.4 ± 1.4
Tip pinch (thumb-little) (Kgf) (mean ± SD)	1.0 ± 0.8	1.1 ± 0.6	1.0 ± 0.5
Grip power (Kg) (mean ± SD)	15.3 ± 7.1	19.5 ± 9.0	19.6 ± 8.8
Pain score (visual analogue score (VAS))			
At entry point (mean ± SD)	46.9 ± 28.8^*∗∗*^	21.7 ± 25.0	29.8 ± 23.9
Most severe pain during 4 weeks (mean ± SD)	72.0 ± 23.4^*∗∗*^	40.7 ± 35.0	56.9 ± 29.8
Average pain during 4 weeks (mean ± SD)	44.9 ± 26.3^*∗∗*^	23.9 ± 25.5	34.8 ± 32.4
Night pain (mean ± SD)	41.8 ± 35.0	25.8 ± 30.7	14.0 ± 26.3
Night pain (existence, nonexistence) (*n* (%))	18 (69), 8 (31)^*φ*^	38 (51), 37 (49)	2 (25), 6 (75)
DASH score	25.0 ± 17.5	28.5 ± 20.4	38.8 ± 26.5
*The data at 12 weeks after surgery*			
Distal latency (ms) (mean ± SD)	7.1 ± 3.9	7.9 ± 4.0	7.2 ± 4.1
S-W test (mean ± SD)	3.8 ± 1.0	4.0 ± 0.9	3.7 ± 1.3
MMT of APB (mean ± SD)	3.9 ± 0.7	3.8 ± 0.7	3.5 ± 0.8
Tip pinch (thumb-index) (Kgf) (mean ± SD)	3.6 ± 1.5	3.9 ± 1.6	3.0 ± 1.3
Tip pinch (thumb-little) (Kgf) (mean ± SD)	0.8 ± 0.5	0.8 ± 0.5	0.7 ± 0.4
Grip power (Kg) (mean ± SD)	15.9 ± 6.2	16.7 ± 7.0	13.9 ± 5.2
Pain score (visual analogue score (VAS))			
At entry point (mean ± SD)	12.2 ± 15.3	11.4 ± 19.1	28.3 ± 22.2
Most severe pain during 4 weeks (mean ± SD)	32.4 ± 28.6	24.6 ± 25.1^*ψ*^	50.3 ± 26.6
Average pain during 4 weeks (mean ± SD)	13.5 ± 16.2	12.5 ± 18.7	26.8 ± 20.4
Night pain (mean ± SD)	10.5 ± 19.9^*ψ*^	9.6 ± 19.2^*ψψ*^	33.6 ± 32.8
Night pain (existence, nonexistence) (*n* (%))	9 (35), 17 (65)^*φ*^	22 (29), 53 (71)	6 (75), 2 (25)
DASH score	21.9 ± 19.3	21.7 ± 18.4	26.2 ± 26.4

SD: standard deviation; S-W test: Semmes-Weinstein Monofilaments test; MMT: Manual Muscle Testing; APB: abductor pollicis brevis; DASH: Disability of Arm, Shoulder, and Hand; ^*∗*^significantly different to Unchanged group (*p* < 0.05); ^*∗∗*^significantly different to Unchanged group (*p* < 0.01); ^*ψ*^significantly different to Worsened group (*p* < 0.05); ^*ψψ*^significantly different to Worsened group (*p* < 0.01); ^*φ*^*p* < 0.05 calculated by *χ*^2^ test.
